# P-1248. The Effect of Pantoprazole on Doravirine/Islatravir Pharmacokinetics

**DOI:** 10.1093/ofid/ofae631.1430

**Published:** 2025-01-29

**Authors:** Randolph P Matthews, Nancy Kim, Yang Liu, Deanne Jackson Rudd, Graig Garrett, Jocelyn Gilmartin, Walter Kraft, Ryan Vargo, S Aubrey Stoch, Marian Iwamoto

**Affiliations:** Merck & Co., Inc., North Wales, Pennsylvania; Merck & Co., Inc., North Wales, Pennsylvania; Merck & Co., Inc., North Wales, Pennsylvania; Merck & Co., Inc., North Wales, Pennsylvania; Merck & Co., Inc., North Wales, Pennsylvania; Merck & Co., Inc., North Wales, Pennsylvania; Thomas Jefferson University, Philadelphia, Pennsylvania; Merck & Co., Inc., North Wales, Pennsylvania; Merck & Co., Inc., North Wales, Pennsylvania; Merck & Co., Inc., North Wales, Pennsylvania

## Abstract

**Background:**

Doravirine/islatravir (DOR/ISL 100/0.25 mg) is a daily, oral, fixed-dose combination being developed for the treatment of HIV infection. As people living with HIV are commonly prescribed antacids or acid-reducing agents, we investigated the effects of pantoprazole, a proton pump inhibitor (PPI), on DOR/ISL pharmacokinetics (PK).

**Methods:**

This was a randomized, open-label substudy in participants without HIV. Single-dose DOR/ISL (100/0.75 mg) was administered, followed by a DOR/ISL washout interval of at least 7 days. During the washout period, pantoprazole 40 mg daily was initiated and administered for 5 days, with DOR/ISL coadministered on day 5. Blood was collected to evaluate ISL and DOR PK. Safety and tolerability were assessed throughout the study.

**Results:**

Twenty-one participants were randomized to receive DOR/ISL; 6 participants completed the DOR/ISL + pantoprazole substudy. Pantoprazole had no clinically meaningful effect on DOR PK after coadministration with DOR/ISL. ISL PK were similar after coadministration of pantoprazole compared with PK after administration of DOR/ISL alone. The geometric least-square mean ratio (GMR) (90% CI) (DOR/ISL + pantoprazole over DOR/ISL) for DOR AUC_0-24_ and C_max_ were 0.93 (0.78-1.11) and 0.98 (0.80-1.20), respectively. The GMR (90% CI) for ISL AUC_0-24_ and C_max_ were 1.05 (0.94-1.16) and 0.99 (0.72-1.35), respectively. DOR/ISL administered alone or in combination with pantoprazole was generally well tolerated. Of participants who received DOR/ISL alone or with pantoprazole, 24% (5/21) had ≥ 1 adverse event (AE). No AE was reported more than once. There were no serious AEs, and one participant discontinued due to an AE (abdominal pain thought to be secondary to pantoprazole).

**Conclusion:**

Pantoprazole had no clinically meaningful effect on the PK of DOR, and ISL PK was similar after coadministration with pantoprazole, supporting the use of DOR/ISL with acid-reducing agents. The combination was generally well tolerated.

**Disclosures:**

**Randolph P. Matthews, MD, PhD**, Merck & Co., Inc.: Employee **Nancy Kim, MD**, Merck & Co., Inc.: Employee **Yang Liu, PhD**, Merck & Co., Inc.: Employee **Deanne Jackson Rudd, PhD**, Merck & Co., Inc.: Employee **Graig Garrett, BS**, Merck & Co., Inc.: Employee **Jocelyn Gilmartin, BS**, Merck & Co., Inc.: Employee **Ryan Vargo, PhD**, Merck & Co., Inc.: Employee **S. Aubrey Stoch, MD**, Merck & Co., Inc.: Employee **Marian Iwamoto, MD, PhD**, Merck & Co., Inc.: Former employee

